# Structure-Based Virtual Screening, ADMET Properties Prediction and Molecular Dynamics Studies Reveal Potential Inhibitors of *Mycoplasma pneumoniae* HPrK/P

**DOI:** 10.3390/life14060657

**Published:** 2024-05-22

**Authors:** Shen Li, Ying Zhou, Yujuan Yan, Yinying Qin, Qilu Weng, Litao Sun

**Affiliations:** 1 School of Public Health (Shenzhen), Shenzhen Campus of Sun Yat-Sen University, Sun Yat-Sen University, Shenzhen 518107, China; lish376@mail2.sysu.edu.cn (S.L.); zhouy689@mail2.sysu.edu.cn (Y.Z.); yanyj29@mail2.sysu.edu.cn (Y.Y.); qinyy8@mail2.sysu.edu.cn (Y.Q.); wengqlu@mail2.sysu.edu.cn (Q.W.); 2 Shenzhen Key Laboratory of Pathogenic Microbes and Biosafety, Shenzhen 518107, China

**Keywords:** HPrK/P, discovery of inhibitors, virtual screening, molecular docking, molecular dynamics (MDs), *Mycoplasma pneumoniae*

## Abstract

*Mycoplasma pneumoniae* pneumonia (MPP) is a frequent cause of community-acquired pneumonia (CAP) in children. The incidence of childhood pneumonia caused by *M. pneumoniae* infection has been rapidly increasing worldwide. *M. pneumoniae* is naturally resistant to beta-lactam antibiotics due to its lack of a cell wall. Macrolides and related antibiotics are considered the optimal drugs for treating *M. pneumoniae* infection. However, clinical resistance to macrolides has become a global concern in recent years. Therefore, it is imperative to urgently identify new targets and develop new anti-*M. pneumoniae* drugs to treat MMP. Previous studies have shown that deficiencies in HPrK/P kinase or phosphorylase activity can seriously affect carbon metabolism, growth, morphology, and other cellular functions of *M. pneumoniae*. To identify potential drug development targets against *M. pneumoniae*, this study analyzed the sequence homology and 3D structure alignment of *M. pneumoniae* HPrK/P. Through sequence and structure analysis, we found that HPrK/P lacks homologous proteins in the human, while its functional motifs are highly conserved in bacteria. This renders it a promising candidate for drug development. Structure-based virtual screening was then used to discover potential inhibitors among 2614 FDA-approved drugs and 948 bioactive small molecules for *M. pneumoniae* HPrK/P. Finally, we identified three candidate drugs (Folic acid, Protokylol and Gluconolactone) as potential HPrK/P inhibitors through molecular docking, molecular dynamics (MDs) simulations, and ADMET predictions. These drugs offer new strategies for the treatment of MPP.

## 1. Introduction

*Mycoplasma pneumoniae* is a significant cause of respiratory infections in school-age children globally, responsible for 20–40% of community-acquired pneumonia cases in this age group. Approximately 10% of infected children ultimately develop infectious pneumonia, with a global epidemic peak occurring every 3–7 years [1,2,3,4]. *M. pneumoniae* is transmitted through airborne droplets, and the number of infections worldwide has increased rapidly [5,6,7]. It is important to note that there is currently no effective vaccine for *M. pneumoniae* [4]. *M. pneumoniae* is recognized as a leading cause of respiratory diseases in humans [8]. It is a small prokaryotic microorganism that lacks a cell wall and only has a cell membrane. Its genome is approximately 816 kb in size and encodes 688 predicted ORFs [9,10]. The attachment organelles consist mainly of P1, P30, P40, P90, and high-molecular-weight protein 1 (HMW1) and 3 (HMW3). The P1 protein is an immunodominant protein that mediates *M. pneumoniae* adhesion to host cell membranes. It can directly bind to membrane surface receptors [11,12]. The CARDS toxin, encoded by gene MPN372, is a unique bacterial ADP-ribosylation and vacuolar toxin that induces Community-Acquired Respiratory Distress Syndrome (CARDS) [13]. The gene MPN400 encodes IbpM, an immunoglobulin-binding protein that strongly binds to various immunoglobulins (IgM, IgG, and IgA) produced by the host, thereby evading the host’s immune responses [14]. In *M. pneumoniae* metabolism, HPrK/P, the HPr kinase/phosphatase, can phosphorylate and dephosphorylate HPr, the histidine-containing protein, at residue serine 46. Phosphorylated HPr, as part of the phosphotransferase system (PTS), regulates carbohydrate transport and metabolism [15,16]. In comparison to eukaryotic Ser/Thr protein kinases, the sequence of *M. pneumonic* HPrK/P exhibits lower homology with eukaryotic Ser/Thr protein kinases. It lacks the highly conserved sequence motif that is characteristic of the eukaryotic protein kinase family. Instead, it contains the walker motif A (G/AxxxxGKT/S), which is present in many nucleotide-binding proteins and forms a typical ring [17], called P-Loop, that binds the phosphate group of ATP. The presence of the walker motif in HPrK/P suggests that it belongs to a new family of Ser/Thr protein kinases.

*M. pneumoniae* infection is typically self-limiting. Antibiotics can shorten the disease course and reduce the occurrence of complications. Tetracycline and fluoroquinolone antibiotics are effective against *M. pneumoniae* infection [18]. However, tetracycline drugs may cause bone and teeth discoloration in young children, and fluoroquinolones may affect muscles, joints, and tendons. Macrolide antibiotics have been the preferred treatment for *M. pneumoniae* infections due to their lower incidence of side effects [19]. *M. pneumoniae* lacks a cell wall, making it naturally resistant to beta-lactam antibiotics. However, the increasing prevalence of macrolide-resistant *M. pneumoniae* strains poses a challenge in treating *Mycoplasma pneumoniae* pneumonia (MPP) [20]. Epidemiological studies have shown that the isolation rate of macrolide-resistant *M. pneumoniae* strains has been increasing over the years. In some areas of southeast Asia, the resistance rate can reach 80 percent in some areas [20,21]. The emergence of antibiotic resistance is a significant global challenge in the treatment of *Mycoplasma pneumoniae* infections.

Previous studies have shown that deficiencies in HPrK/P kinase or phosphorylase activity can significantly affect carbon metabolism, growth, morphology and other cellular functions of *Staphylococcus xylosus*, *Lactobacillus casei*, macrolide-resistant *Mycoplasma pneumoniae* and *Bacillus subtilis* [22,23,24,25]. Therefore, bacterial kinases are also receiving increasing attention as promising targets for antimicrobial drug discovery [26,27,28]. In this study, the HPrK/P of *M. pneumoniae* was used as a target to screen inhibitors. We identified the potential active pockets of HPrK/P through previous studies and structural analysis. A library of FDA-approved small molecule drugs and 948 bioactive small molecules has been identified through virtual screening methods, and the interactions of drug candidates with HPrK/P have been studied through molecular dynamics (MDs) simulations. Our results show that Folic acid, Protokylol and Gluconolactone are potential kinase inhibitors of *M. pneumoniae* HPrK/P, which may contribute to the development of a new class of therapeutic drugs and provide a new strategy to solve the problem of drug resistance in MMP clinical treatment.

## 2. Materials and Methods

### 2.1. Analysis of HPrK/P Sequences

Sequences were retrieved from UniProt (https://www.uniprot.org/, accessed on 15 January 2024). The sequences used in this study are summarized in Table S1, including 3 *M. pneumoniae* HPrK/P sequences, 17 bacterial HPrK/P sequences, 2 human BRSK sequences, and 2 mouse BRSK sequences. The phylogenetic tree was constructed with only synonymous substitutions that were used to identify differences by the neighbor-joining (NJ) method in Geneious Prime [29]. Evolutionary genetic distances among amino acid sequences were estimated by the Jukes–Cantor method using Geneious Prime2023.2 software. The tree was edited in another online software Evolgenius-v2 (https://evolgenius.info/evolview-v2, accessed on 2 December 2023). Multiple sequence alignment was performed using ClustalW (https://www.ebi.ac.uk/Tools/msa/clustalo/, accessed on 2 December 2023) and the sequences were further analyzed using ESPript3.0 (https://espript.ibcp.fr/ESPript/ESPript/index.php, accessed on 2 December 2023).

### 2.2. Preparation of Target Protein

The structure of *M. pneumoniae* HPrK/P was obtained from the Protein Data Bank (PDB ID: 1KNX). We compared the amino acid sequences of HPrK/P in different bacteria by multiple sequence alignment (MSA). And we analyzed the data of the effect of *M. pneumoniae* HPrK/P amino acid mutation on enzyme activity in the UniProt database (https://www.uniprot.org/, accessed on 2 November 2023). And we utilized Schrödinger’s SiteMap to predict the potential binding site, which suggested the highest drug ability score. Finally, we selected the active pocket corresponding to the enzyme activity experiment and structural analysis. The coordination of the active sites grid box was set to encompass the entire binding pocket as defined by the structure and SiteMap analysis, with a buffer zone to allow for flexibility in ligand positioning. Docking/Grid Box Coordinate: The centroid of the grid box was set at (45.4, 69.6, 0.9), with dimensions (15 Å, 15 Å, 15 Å) angstroms to ensure complete coverage of the potential interaction space. The pocket was used as a potential binding pocket for small molecule inhibitors and pretreated with Schrodinger’s Protein Preparation Module. This process included the elimination of water molecules and unwanted ions, the completion of missing side chains and circulating regions, and energy minimization. Subsequently, the docking grid file was generated by Schrödinger’s Glide Grid (Release 2019-2, Schrödinger LLC, New York, NY, USA, 2019) module for subsequent molecular docking studies.

### 2.3. Construction of Small Molecular Ligands

The small-molecule database is derived from the FDA-approved small-molecule database (2614) from DrugBank (Table S3) and the 948 bioactive small molecules (Table S4) built by our laboratory. Small molecules were treated by Schrödinger’s LigPrep module (Release 2019-2, Schrödinger LLC, New York, NY, USA, 2019), including protonation, desalination, hydrogenation, generation of tautomers and stereoisomers, and energy minimization [30]. Minimization/Iteration Step: The protein–ligand complex structures were subjected to energy minimization using the OPLS3e force field. The iterative process continued until the convergence criterion of a root mean square (RMS) gradient of 0.01 kcal/mol/Å was achieved. Charge Type(s): Charges were assigned using the Schrödinger suite’s Maestro software2017, implementing the OPLS3e force field, which accounts for partial atomic charges. Non-Polar Hydrogens: Non-polar hydrogens were merged, and their electronic effects were distributed to the bonding heavy atoms, following the standard protocol within the Schrödinger’s LigPrep module. Forcefield: As mentioned, the OPLS3e force field was utilized for all minimization and molecular dynamics simulations. These treated small molecules were used for subsequent docking studies.

### 2.4. High-Throughput Virtual Screening (HTVS)

The first round of virtual screening was performed by Schrödinger’s Glide module in the high-throughput virtual screening (HTVS) mode, and the top 20% of the molecules were retained before standard precision (SP) screening. Similarly, the top 20% of molecules from the SP screening were retained for the final Extra-Precision (XP) screen. The 20% of molecules with the highest scores in the XP screening underwent MM/GBSA analysis and calculated the binding free energy, and the top 10 molecules were selected based on the MM/GBSA_∆Gbind_ scores for binding mode analysis.

### 2.5. Toxicity Prediction

Assessing the Absorption, Distribution, Metabolism and Excretion (ADME) properties of drugs is one of the important steps in the drug development process. An ideal inhibitor should be effective and have little or no toxicity. Drugs strongly bound to *M. pneumoniae* HPrK/P selected from a virtual screening were examined via the SwissADME web platform (http://www.swissadme.ch/, accessed on 12 January 2024) and admetSAR web platform (http://lmmd.ecust.edu.cn/admetsar2, accessed on 12 January 2024) for toxicity assessment. Two different levels of toxicity were tested: AMES Toxicity and Carcinogenicity.

### 2.6. Molecular Dynamics (MDs) Simulation Analysis

GROMACS2022.3 version software was used for molecular dynamics simulations [31,32]. For small molecule preprocessing, AmberTools22 was used to add the GAFF force field to small molecules [33], and Gaussian 16 W was used to hydrogenate small molecules and calculate RESP potential. The potential data would be added to the molecular dynamics system topology file. The simulation conditions were carried out at a static temperature of 300 K and at atmospheric pressure (1 Bar), the force field was Amber99sb-ildn, the solvent was water molecule (Tip3p water model) and the total charge of the simulated system was neutralized by adding an appropriate number of Na^+^ ions. The molecular dynamics simulation system first adopted the steepest descent method to minimize the energy, and then carried out the isothermal isovolumetric ensemble (NVT) equilibrium and constant-pressure, constant-temperature (NPT) equilibrium for 100,000 steps, respectively, with the coupling constant of 0.1 ps and the duration of 100 ps. Finally, the free molecular dynamics simulation was performed. The process consisted of 50,000,000 steps with a step length of 2 fs and a total duration of 100 ns. After the simulation, the trajectory was analyzed with the built-in tool of the software, and the root-mean-square deviation (RMSD), root-mean-square fluctuation (RMSF) and protein rotation radius of each amino acid movement trajectory were calculated. Additionally, the total free energy of binding was estimated using the MM-GBSA method, which took into account various energy components such as molecular mechanics energy, polar and nonpolar solvation free energy contributions, and entropy terms.

### 2.7. Free Binding Energy (MM/GBSA) Analysis

The MM/GBSA method is often used to calculate the binding free energy of ligands that bind to receptors. In order to calculate the average of all ensembles for affinity calculation, the MM/GBSA method usually extracts structures at different time points from a single trajectory of the complex, or from three trajectories of the complex, receptor, and ligand, respectively. The binding free energy of protein–ligand complexes (HPrK/P–compounds 1, 2, 3, 4, 5, 6) were calculated using the g_mmpbsa tool [34]; g_mmpbsa calculated the binding free energy of complex structure using the Generalised-Born and surface-area solvation (MM/GBSA). We calculated the free energy from MD trajectories of 100 ns duration, sampling snapshots every 100 ps. The binding free energy was calculated as the sum of van der Waal energy, electrostatic energy, polar solvation energy, and the solvent accessible surface area (SASA) energy. The calculation method is as follows:

**             ΔG_binding_ = G_complex_ − G_receptor_ − G_ligand_** **     ΔG_GAS_ = ΔG_VDWAALS_ + ΔE_EL_** **    ΔG_SOLV_ = ΔE_GB_ + ΔE_SURP_** **      ΔTotal = ΔG_GAS_ + ΔG_SOLV_** **ΔG_MM-GBSA_ = ΔTotal**

## 3. Results

### 3.1. M. pneumoniae HPrK/P Has Conserved Active Sites with Bacterial HPrK/P and Shares Low Homology with Human Serine/Threonine Protein Kinases

**Figure 1:**
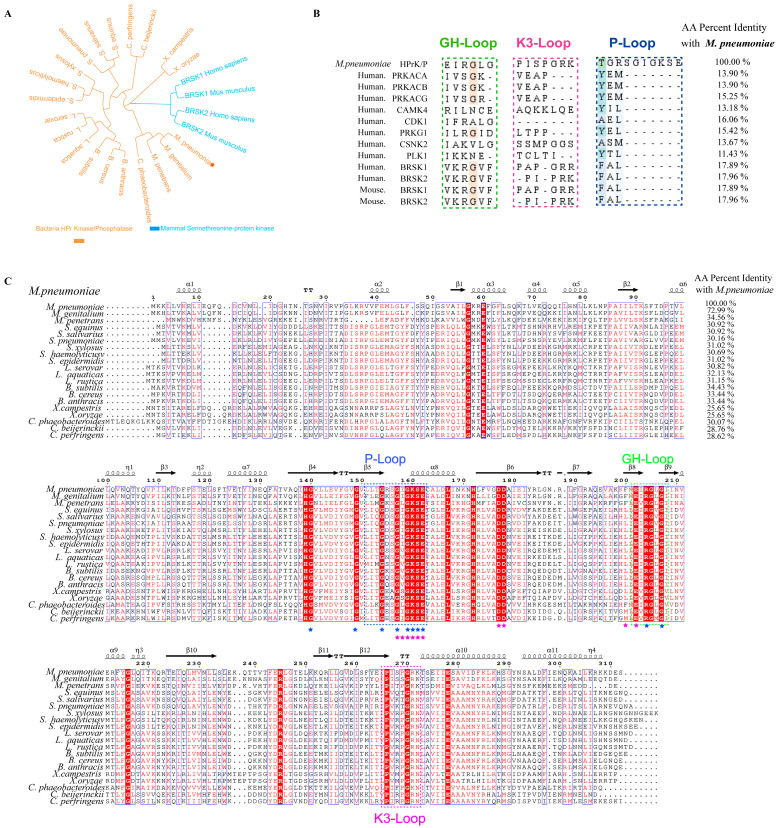
Homologous analysis of *M. pneumoniae* and bacterial HPrK/P sequences. (**A**) Phylogenetic relationship of human, Mycoplasma and bacterial HPrK/P sequences. The phylogeny of the amino acid sequences was estimated by the neighbor-joining method for the HPrK/P of Mycoplasma and bacteria, serine/threonine kinase proteins (BRKPs) for the human and mouse deposited in the UniProt database (Table S1). (**B**) Sequence alignment of *M. pneumoniae* HPrK/P and various human serine–threonine protein kinase families. (**C**) Alignment of bacterial HPrK/P sequences. The secondary structure at the top is defined by DSSP for the *M. pneumoniae*. The highly conserved sites in the enzyme activity test are marked with blue stars, and the highly conserved sites in the docking interaction analysis are marked with pink stars.

To evaluate whether *M. pneumoniae* HPrK/P could be a suitable target for drug specificity, we analyzed the sequence and structural characteristics of *M. pneumoniae*. Firstly, we compared the sequence homology of *M. pneumoniae* HPrK/P with bacterial HPrK/P and human serine/threonine protein kinase (BRSK). We used 17 bacterial HPrK/P sequences, 2 human BRSK sequences, and 2 mouse BRSK sequences (Table S1) for homology comparison. The phylogenetic tree is shown in Figure 1A. The blue branches represent the mammalian serine/threonine protein kinase family, and the orange branches represent the bacterial and Mycoplasma HPrK/P families. The phylogenetic tree indicates that the mammalian BRSK family, bacterial HPrK/P and *M. pneumoniae* HPrK/P sequences belong to four different families. It is worth noting that most bacteria belong to the same branch while *M. pneumoniae* and *Xanthomonas campestris* are in independent branches. This suggests that HPrK/P shares evolutionary homology among different bacteria. To assess the homology between Mycoplasma and human serine/threonine protein kinases, we curated nine representative sequences from the human serine/threonine family (Table S1). Through multiple sequence alignment, illustrated in Figure S1A, it is evident that Mycoplasma and various human serine/threonine family sequences exhibit minimal similarity, with the maximum similarity observed at only 17.96% (BRSK2). Furthermore, no analogous motifs, including the highly conserved functional motifs (P-loop, GH-loop, and K3-loop), were identified in HPrK/P (Figure 1B). The structural alignment of the active pockets further underscores the lack of similarity between HPrK/P and human serine/threonine protein kinases (Figure S1B).

Furthermore, *M. pneumoniae* has retained its evolutionary specificity, as demonstrated by the sequence alignment results depicted in Figure 1C. Multiple sequence alignments revealed that the HPrK/P sequence similarity among different bacteria is approximately 30%, indicating a low degree of sequence similarity across bacterial species. Notably, *M. pneumoniae* shares conserved regions such as the GH-loop, P-loop, and K3-loop with diverse bacterial HPrK/P sequences. The amino acid sequences within these regions exhibit high conservation. Importantly, these regions have been identified as functional domains in HPrK/P of *B. subtilis*, *L. casei*, and *S. xylosus*. This suggests that *M. pneumoniae* and various bacterial HPrK/P variants may share similar kinase and phosphatase catalytic mechanisms. In summary, our homology analysis and sequence alignment support the notion that *M. pneumoniae* HPrK/P holds promise as a target for drug design and discovery due to its evolutionary specificity in discriminating between humans and bacteria, as well as its broad-spectrum potential.

### 3.2. The Location of the Active Pocket in M. pneumoniae HPrK/P Is Situated in the Space between GH-Loop and P-Loop

**Figure 2:**
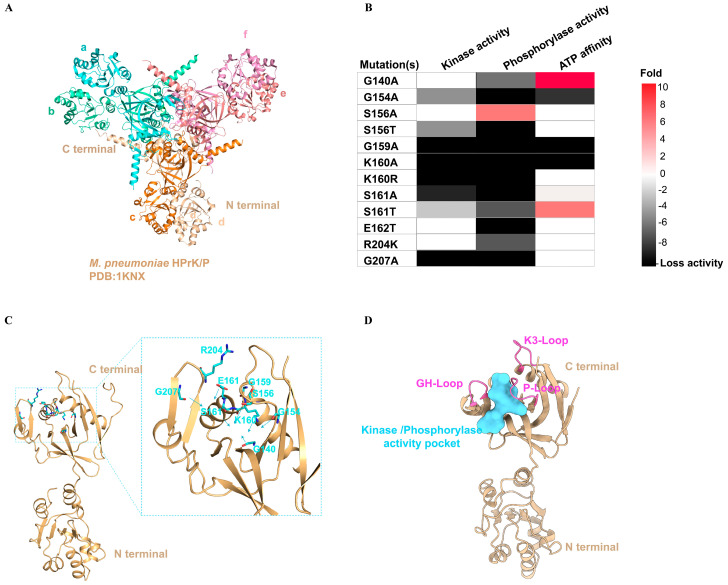
The catalytic pocket of *M. pneumoniae* HPrK/P for virtual screening. (**A**) Overview of the hexamer structure of *M. pneumoniae* HPrK/P (PDB ID: 1KNX). (**B**) Effect of conserved amino acid mutations on enzyme activity (data from UniProt database). (**C**) The conserved residues associated with enzyme activity are shown in the form of a stick. The direction of the arrows represents the direction of the side chains of these residues. (**D**) The kinase/phosphorylase pocket is shown on the surface. The P-loop, GH-loop and K3-loop are colored hot pink.

The crystal structure of *M. pneumoniae* HPrK/P has been solved by Gregory S. Allen et al. (Figure 2A). The structural analysis reveals that *M. pneumoniae* HPrK/P shares similar tertiary and quaternary structures with the HPrK/P crystal structures observed in *L. casei* [35] and *S. xylinum* [36]. *M. pneumoniae* HPrK/P is a homohexamer with approximately 300 residues folded into two distinct domains. The C-terminal domain of HPrK/P exhibits both kinase and phosphorylase activities, whereas the function of the N-terminal domain remains undetermined. Notably, the C-terminal domain contains a Walker A motif that forms the phosphate-binding loop (P-loop) of the nucleotide binding site. Although the fold of HPrK/P is distinct from eukaryotic protein kinases, it is similar to the small molecule kinases of the P-loop protein family [37]. Therefore, HPrK/P is a member of a new family of protein kinases [38]. Although the crystal structure of *M. pneumoniae* HPrK/P has been solved, the complex structure of HPrK/P and the substrate ATP has not been obtained and the catalytically active pocket of HPrK/P is unknown. In a previous study by K. Steinhauer et al., they identified the key residues that affect HPrK/P phosphorylation, dephosphorylation and ATP binding ability of *M. pneumoniae* HPrK/P through amino acid mutations [24]. The mutation of the residues Gly140, Gly154, Gly159, Lys160, and Ser161 had a significant effect on ATP binding. The mutation of the residues Gly154, Ser156, Gly159, Lys160, Ser161, and Gly207 also significantly affected the kinase activity of HPrK/P (Figure 2B). Interestingly, these residues (Marked with a blue star in Figure 1C) are highly conserved in *M. pneumoniae* and bacterial HPrK/P sequences. This indicates that the bacterial and *M. pneumoniae* HPrK/Ps may share a conserved catalytic mechanism of kinase activity. Through structural analysis, we found that these highly conserved residues are located in the P-loop and GH-loop regions. By further analyzing the direction of the side chains of these conserved amino acids, we found that they all point to the space situated between the P-loop and GH-loop (Figure 2C). This indicates that the kinase activity pocket of *M. pneumoniae* HPrK/P is located in this region (Figure 2D). This potential kinase activity pocket was used as a binding pocket for screening inhibitor candidates.

### 3.3. Virtual Screening of Small Molecules with M. pneumoniae HPrK/P

**Figure 3:**
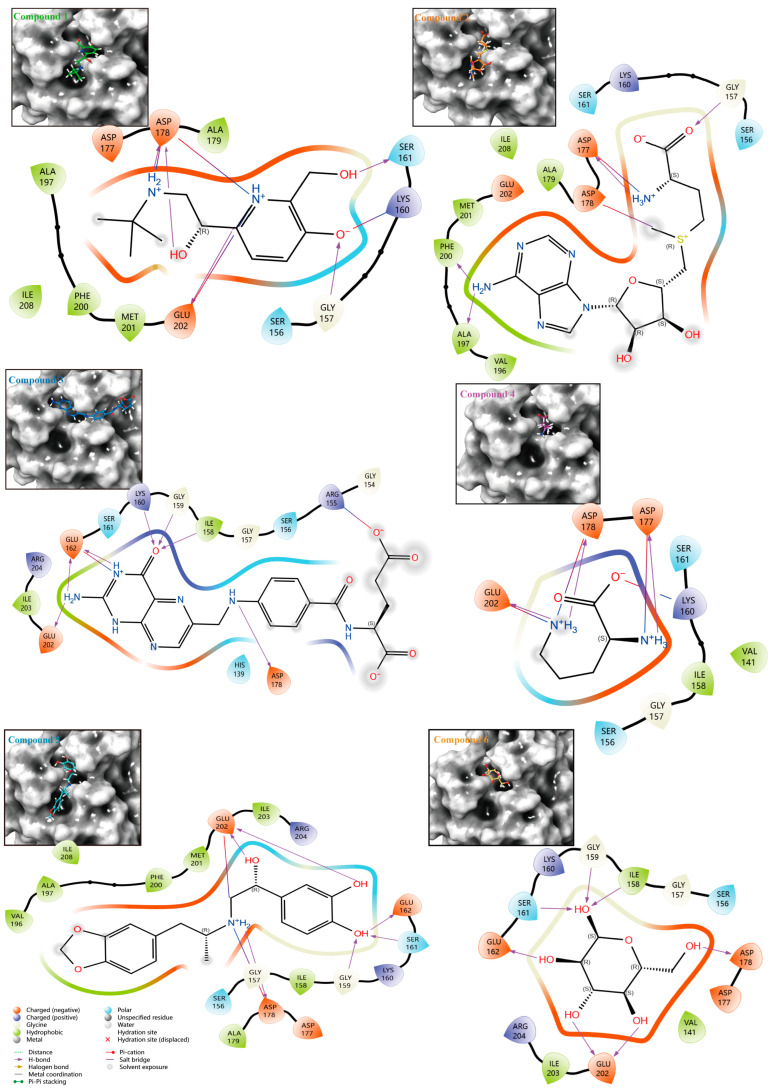
Three-dimensional and two-dimensional interactions between HPrK/P and the top six potential inhibitors.

**Figure 4:**
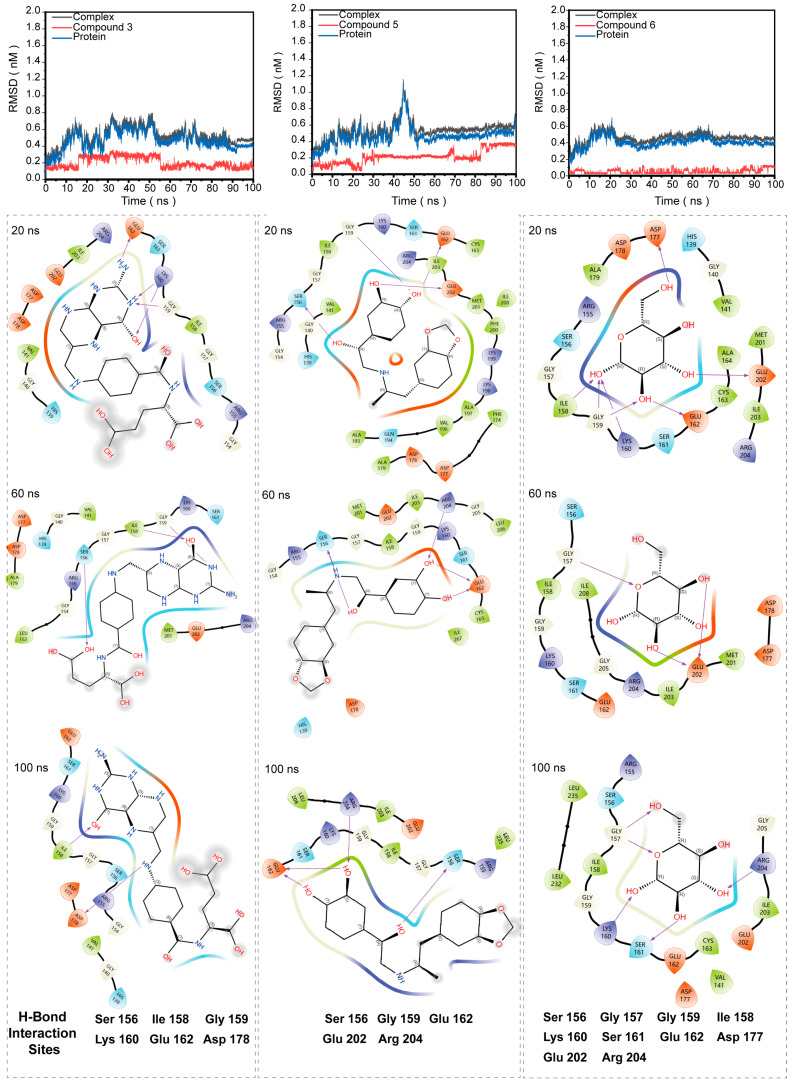
Analysis of the interaction between compounds 3, 5, 6 and protein in a molecular dynamic simulation. Conformational snapshots were extracted from the simulation at 20 ns, 60 ns, and 100 ns, and a 2D interaction plot was generated. The residues involved in hydrogen bond formation are listed statistically on the right side of the diagram.

To rapidly screen candidate inhibitors of HPrK/P, we completed a targeted virtual screening of 2614 FDA-approved drugs and 948 bioactive small molecules for HPrK/P using Schrödinger’s Computational Platform for Molecular Discovery (Release 2019-2, Schrodinger LLC, New York, NY, USA, 2019) to achieve ‘new use of old drugs’ and avoid drug toxicity and unclear targets. Finally, we selected the top 10 small molecules (Table 1) based on the MM/GBSA binding free energy. And then, we performed protein–ligand interaction analysis on the docking results of the top six small molecules (Figure 3). It is obvious that the top six compounds docked into the same active pocket. We systematically analyzed the protein–ligand interaction network and counted the number of interactions between each compound and protein amino acids. Compound 1 exhibits the formation of five hydrogen bonds with amino acids Gly157, Ser161, Asp178, and Glu202. Compound 2 establishes three hydrogen bonds with amino acids Gly157, Asp177, and Phe200. In the case of Compound 3, six hydrogen bonds are observed with amino acids Ile158, Lys160, Glu162, Asp178, and Glu202. Compound 4 forms three hydrogen bonds with amino acids Asp177, Asp178, and Glu202. Compound 5 demonstrates the involvement of six hydrogen bonds with amino acids Gly159, Ser161, Glu162, Asp178, and Glu202. Lastly, Compound 6 engages in six hydrogen bonds with amino acids Ile158, Gly159, Ser161, Glu162, Asp178, and Glu202. In addition, the top six compounds formed multiple hydrophobic, salt bridge and polar interactions with other amino acid residues (Table 2). It is worth mentioning that most of the residues that form hydrogen bonds and interact with the top six compounds are located on the P-loop and GH-Loop. Although the other amino acids are not located on the P-loop and GH-loop, such as Val141, Ile158, Asp177, Asp178, Glu202, Ile208, these amino acid residues are also highly conserved in *M. pneumoniae* and bacterial HPrK/P (Marked with a pink star in Figure 1C). In addition, we analyzed the hydrogen bond interaction sites of small molecules 3, 5, 6 with HPrK/P in a molecular dynamics simulation, and selected three complex trajectories at 20 ns, 60 ns, and 100 ns (Figure 4). The results show that the amino acids which form hydrogen bonds with HPrK/P are also highly conserved in molecular dynamics simulation. This suggests that these small molecule candidates may not only be inhibitors of *M. pneumoniae* HPrK/P kinases, but also have potential as broad-spectrum inhibitors of bacterial HPrK/P kinases.

### 3.4. The ADME Properties and Toxicity Testing

Assessing the pharmacological properties of lead candidates is a crucial step in drug development. We predicted the ADMET properties of the top 10 compounds (Table S2) by two web platforms (SwissADME and admetSAR). These parameters included water solubility, GI absorption, BBB permeant, Pgp substrate, cytochrome P450 inhibitor, skin penetration ability (logKp), AMES test, and carcinogenicity. As we expected, the candidate compounds screened based on the FDA-approved drug library and 948 bioactive small molecules demonstrated good safety and bioavailability. The compound 6 is from the natural product database, which has good absorption and safety.

### 3.5. Molecular Dynamics Simulations of the Top Six Protein–Ligand Complexes

**Figure 5:**
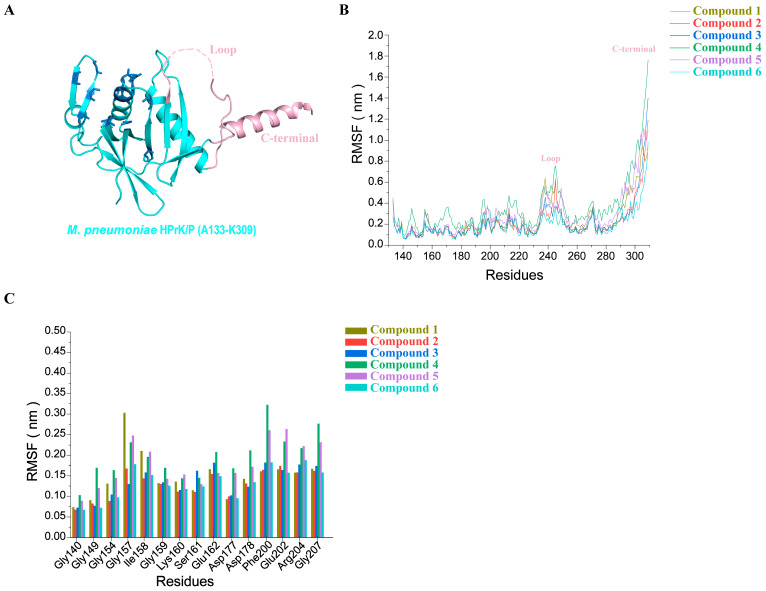
The MD simulation (RMSF analysis) of HprK/P–compound 1, HprK/P–compound 2, HprK/P–compound 3, HprK/P–compound 4, HprK/P–compound 5, HprK/P–compound 6 complexes for 100 ns. (**A**) The C-terminal domain of *M. pneumoniae* HPrK/P shown in the cartoon. The residues that form an H-bond with the top six compounds are shown in the form of a blue stick. (**B**) The RMSF analysis of the top six complexes. The loop and C-terminal regions are colored pink (Figure 5A). (**C**) The RMSF analysis of the highly conserved residues that form an H-bond with the top six compounds.

Molecular dynamics simulations can help us to analyze the structural dynamics, conformational fluctuation, and stability of protein–ligand complexes. To verify the flexibility of the complexes formed by six small molecules with HPrK/P, these complexes were further subjected to molecular dynamics simulation analysis. The molecular dynamics simulation was carried out for 100 ns to analyze the flexibility and rigidity of the protein–ligand complexes. The flexibility of the whole complex (HPrK/P–compound 1, HPrK/P–compound 2, HPrK/P–compound 3, HPrK/P–compound 4, HPrK/P–compound 5, and HPrK/P-compound 6) was analyzed based on the RMSD (Figure S2). The conformational fluctuation of the complex was analyzed based on the RMSF (Figure 5), radius of gyration (Rg) (Figure S3) and hydrogen bonds (Figure S4). RMSD reflects the deviation from the initial conformation to the final conformations of proteins, ligands and complexes in the whole 100 ns simulation process. The results show that the ligands have low conformational flexibility in the six complexes during the whole simulation process. The significant conformational fluctuations were observed in the protein of complexes 1, 2, and 4, and the protein–ligand complexes appeared to disassemble in the later stages of the MD simulations. In contrast, the fluctuation of complexes 3, 5, and 6 is minimal. Complexes 3 and 5 exhibit low flexibility after 50 ns, with complex 6 showing the lowest flexibility, reaching stability after only 20 ns. We repeated the molecular dynamics simulation results of complexes 3, 5, and 6. The results showed that the two molecular dynamics simulation results of these three complexes were consistent, indicating that these three small molecules formed stable complexes with HPrk/P (Figure S5). Through molecular dynamics simulations, we extracted the conformations of complexes 3, 5, 6 at 20 ns, 60 ns, and 100 ns. And then, we analyzed the hydrogen bond interaction residues between HPrK/P and compounds 3, 5, 6. We found that these residues are highly conserved among these hot sites in the HPrK/P kinase active pocket. Interestingly, these conserved sites are highly consistent with docking complexes. These results suggest that these amino acids (Ser156, Gly159, Ile158, Lys160, Glu162 and Arg204) are important residues in the kinase active pocket of HPrK/P (Figure 4).

Additionally, we calculated the gyration between proteins and small molecules and created a gyration chart (Figure S3). The results indicate that the combination of HPrK/P with compounds 3, 5, and 6 leads to a gradual decrease in the Rg value, ultimately resulting in stable complexes. The stability of the complexes formed with these three compounds is higher than of those formed with other compounds, as confirmed by RMSD and Gyrate analysis. This finding is consistent with the MM-GBSA value (Table 3).

The RMSF analysis describes the flexible regions of the protein–ligand complexes and determines the movement of each residue within the complex. A higher RMSF value indicates a more flexible region, such as loops, beta-turns, and random coils. We calculated the RMSF to predict the degree of protein structural changes caused by binding ligands (Figure 5B). The results show that the RMSF value of complex 4 is higher than that of the other complexes. Except for the loop region and the C-terminal region (Figure 5A), the RMSF peak value of complex 3, complex 5, and complex 6 are all below 0.3 nm, which indicates that the binding of these three compounds to *M. pneumoniae* HPrK/P is stable. Interestingly, we analyzed the conserved interaction residues in the docking results, and found that the RMSF values of those highly conserved residues located in P-loop and GH-loop were very low (Figure 5A). The RMSF values of those highly conserved residues in complexes 1–6 were 0.15 nm, 0.13 nm, 0.13 nm, 0.20 nm, 0.18 nm, and 0.13 nm, respectively. These results suggest that the binding of small molecules to HPrK/P stabilizes the kinase pocket. Additionally, we examined the hydrogen bond interactions between the top six compounds and HPrK/P (Figure S4). The stability of the complex generally increases with the number of hydrogen bonds. The HPrK/P–compound 1 complex and HPrK/P–compound 2 complex formed an average of two hydrogen bonds, while HPrK/P–compound 4 formed one hydrogen bond. HPrK/P–compounds 3, 5, and 6 all formed four hydrogen bonds, respectively. Compounds 3, 5, and 6 formed more hydrogen bonds with HPrK/P, indicating a more stable complex.

### 3.6. MM-GBSA Binding Energy Calculation

The binding free energy was calculated as the sum of van der Waal energy (ΔVDWAALS), electrostatic energy (ΔE_EL_), polar solvation energy (ΔE_GB_), and the solvent accessible surface area (SASA) energy(ΔE_SURP_). A more negative value for the binding free energy indicates a stronger affinity of the ligands to the receptor. We determined the free binding energy of the HPrK/P–compound complexes from the trajectory obtained from MD simulation of 100 ns (Table 3). The binding free energy for HPrK/P–compound 3, HPrK/P–compound 5 and HPrK/P–compound 6 is −36.66 ± 2.63 kJ·mol^−1^, −30.99 ± 1.99 kJ·mol^−1^ and −36.07 ± 1.73 kJ·mol^−1^, respectively. The results indicate that all three compounds have a significant binding affinity with HPrK/P. The top six docking results were analyzed by MD simulation, and the three candidates were identified.

## 4. Discussion

*Mycoplasma pneumoniae* is a frequent cause of respiratory infections, and community-acquired pneumonia represents a major disease-related burden. The introduction of non-pharmaceutical interventions (NPIs) against COVID-19 has led to an abrupt end to these epidemics and a significant decline in *M. pneumoniae* detection rates globally [39]. But now, *Mycoplasma pneumoniae* pneumonia has re-emerged and is showing a rapid increase trend [5]. However, macrolide-resistant *Mycoplasma pneumoniae* (MRMP) has emerged worldwide, particularly in East Asia, where the proportion of MRMPI strains in China reaches as high as 69% to 95%. In Japan, the detection rate of MRMP among children ranged from 50% to 90% from 2008 to 2015 [40,41,42]. There is currently no effective vaccine to prevent infection. The treatment of MRMP has become more and more challenging. The development of new drugs is urgent and necessary.

Structure-based virtual screening has been widely used in the screening of antiviral and antibacterial drugs [43,44,45]. This method can screen millions of drug libraries in a short time, which is impossible with traditional high-throughput screening methods. However, this method relies on the selection and confirmation of the active pocket of the protein target. Inaccurate target selection can result in candidates with no biological activity. In addition, the selection of small molecule drug libraries should also consider the feasibility of the next biological verification. In Gram-positive bacteria, HPrK/P regulates the transport of the carbon source required for growth and other essential functions. It also regulates the phosphorylation of HPr protein, implicated in the virulence processes of pathogenic bacteria. Most importantly, deficiency in HPrK/P reduces the growth of bacteria. Unlike Gram-positive bacteria, which have hard cell walls, *M. pneumoniae* is a small microorganism without a cell wall. Drugs targeting *M. pneumoniae* HPrK/P are able to pass through the cell membrane and reach the target location. Therefore, the discovery of inhibitors targeting HPrK/P is of great significance for the clinical treatment of MRMP. In this study, we confirmed that *M. pneumoniae* HPrK/P is a good target for drug development through multiple sequence alignment and structural analysis. Furthermore, FDA-approved drug libraries and 948 bioactive small-molecule self-built drug libraries ensure the safety of candidate drugs and the feasibility of biological verification. The strategy of “new uses for old drugs” can shorten the drug development cycle and reduce costs, thus offering significant advantages in clinical drug research and development [46]. We used a structure-based virtual screening method to screen inhibitors that can target *M. pneumoniae* HPrK/P. These candidates may be useful in the clinical treatment of MRMP.

In silicon screening, involving the docking of molecules from a virtual library into a receptor structure to predict binding scores, is a well-established method for hit and lead discovery, contributing significantly to recent drug discovery successes [47,48]. The docking procedure utilizes molecular mechanics, often in an internal coordinate representation, to rapidly sample the conformational space of fully flexible ligands. This can be achieved through empirical 3D shape-matching approaches [49]. Special emphasis is placed on designing ligand scoring functions to effectively eliminate non-binders, thereby reducing the occurrence of false-positive prediction. Structure-based drug screening can help us find reasonable lead compounds in a short time, but it can bring false positive and false negative results. Optimization algorithms and experimental verification will be an effective strategy to solve this problem. Indeed, it is important to ensure the accuracy of the target protein structure used for virtual screening. This can be achieved, for example, by leveraging high-resolution crystal structures of the drug target. Furthermore, integrating various virtual screening methods, including structure-based screening, the molecular dynamics simulations of drugs, drug–target docking, and drug–protein interaction network analysis, can significantly enhance the specificity of screening. Moreover, validating the outcomes of virtual screening through in vitro and in vivo experiments is essential. These experiments involve assessing the affinity and efficacy of drugs towards other proteins to confirm their specificity and effectiveness.

Although the structure of *M. pneumoniae* HPrK/P has been solved, which is without substrate ATP, we were unable to accurately determine the ATP-binding pocket of *M. pneumoniae* HPrK/P. Through multiple sequence alignment, we found that the homology between *M. pneumoniae* and bacterial HPrK/P sequences is low. But there are some highly conserved amino acid sites. By comparing with other bacterial HPrK/P kinase active sites, we determined the location of the *M. pneumoniae* HPrK/P kinase activity pocket. Interestingly, this kinase activity pocket is conserved in *M. pneumoniae* and bacteria. The amino acid sites involved in ATP binding and kinase activity are also highly conserved. This shows that the small molecule inhibitors we screened for *M. pneumoniae* HPrK/P may have the same interactions with other bacterial HPrK/P. This shows that the drugs screened for the HPrK/P kinase activity pocket have the potential to become broad-spectrum antibacterial inhibitors. Furthermore, we screened some potential HPrK/P inhibitors by virtual docking and molecular dynamics simulations. During the analysis of small-molecule interactions with HPrK/P, we observed that hot sites (e.g., Ser156, Gly157, Ile158, Gly159, Glu162, Asp178, Phe200, Glu202, Arg204, Gly207) exhibited stable interaction patterns in both docking and molecular dynamics simulations, which suggests they could be critical sites for future drug screening and modification. Our study identified the conserved activity pocket of HPrK/P kinase through multiple sequence alignment, structural analysis, docking, and molecular dynamics simulation. These findings hold significance for drug screening and design targeting HPrK/P.

Drug reuse is a promising and effective way to explore the new uses of old drugs, and it accelerates the process of drug discovery. Drugs approved by FDA constitute an ideal database for this purpose. These drugs have clear safety and pharmacokinetic characteristics, and the development risk and cost are low. In addition, since preclinical testing and preparation development have been completed, the time for drug development can be shortened. All in all, repositioning old drugs for new applications can save years of development time and reduce risks and costs. Through virtual screening, ADMET analysis and molecular dynamics simulation, we identified three candidate compounds. Compound 3 is Folic acid, also known as folate or Vitamin B9, is a member of the B vitamin family and an essential cofactor for enzymes involved in DNA and RNA synthesis. Folic acid is a nutrient used to treat megaloblastic anemia and is found in many supplements. Compound 5 is Protokylol, which is a β-adrenergic receptor agonist used as a bronchodilator in Europe and the United States. Compound 6 is Gluconolactone, which is a polyhydroxy acid used in the dissolution of calculi and as an additive in various drug products to maintain consistency and other characteristics. These three drugs are FDA-approved drugs with well-defined targets and safety profiles. Although these three drugs are not among the top three in the molecular docking results, the docking is only a preliminary screening. In MD simulations, these three small molecules showed better stability and higher affinity with the HPrK/P complex. This shows that the results of virtual screening need a further comprehensive evaluation, such as ADMET analysis, MD simulations or activity verification.

Our results show that HPrK/P is a promising target for drug screening and could potentially serve as a broad-spectrum antibacterial drug target, which provides a new strategy to solve the problem of MRMP and bacterial antibiotic resistance in clinic. Through virtual screening and molecular dynamics simulation, we have identified three *M. pneumoniae* HPrK/P inhibitors. In conclusion, *M. pneumoniae* HPrK/P represents a promising target for drug screening, with the potential for designing broad-spectrum antibacterial agents. Folic acid, Protokylol and Gluconolactone are identified as potential inhibitors targeting *M. pneumoniae* HPrK/P. These three candidates may provide new options for the clinical treatment of MRMP.

## Figures and Tables

**Table 1:** List of MM-GBSA scores for the top 10 compounds.

No.	Database ID	CAS Number	Structure	MMGBSA dGBind (kcal/mol)
1	DB01291	38677-81-5	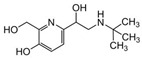	−57.38
2	DB00118	29908-03-0	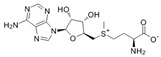	−56.52
3	DB00158	59-30-3	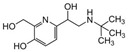	−45.95
4	DB00129	70-26-8	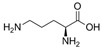	−43.72
5	DB06814	136-70-9	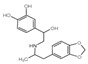	−42.96
6	T0933	90-80-2	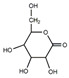	−42.74
7	DB01288	13392-18-2	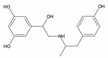	−41.08
8	DB01362	66108-95-0	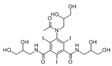	−35.75
9	DB06603	404950-80-7	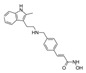	−35.43
10	DB01102	128470-16-6	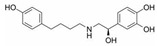	−34.96

**Table 2:** List of the residues and types of intermolecular interactions involved when HPrK/P is docked with the top six compounds.

Complex	H-Bond		Hydrophobic	Salt Bridge	Polar	Positive	Negative
	Residues	Number	Residues	Residues	Residues	Residues	Residues
HPrK/P-Compound 1	Gly157	1	Ala197	Lys160	Ser156	Lys160	Asp177
	Ser161	1	Phe200	Asp178	Ser161		Asp178
	Asp178	2	Met201				Glu202
	Glu202	1	Ile208				
HPrK/P-Compound 2	Gly157	1	Ala179	Asp177	Ser156	Lys160	Asp177
	Asp177	1	Val196	Asp178	Ser161		Asp178
	Phe200	1	Ala197				Glu202
			Phe200				
			Met201				
HPrK/P-Compound 3	Ile158	1	Ile158	Arg155	His139	Arg155	Glu162
	Lys160	1	Ile203	Glu162	Ser161	Lys160	Asp178
	Glu162	2				Arg204	Glu202
	Asp178	1					
	Glu202	1					
HPrK/P-Compound 4	Asp177	1	Val141	Lys160	Ser156	Lys160	Asp177
	Asp178	1	Ile158	Asp177	Ser161		Asp178
	Glu202	1		Asp178			Glu202
				Glu202			
HPrK/P-Compound 5	Gly159	1	Ile158	Asp178	Ser156	Lys160	Asp177
	Ser161	1	Ala197	Glu202	Ser161	Arg204	Asp178
	Glu162	1	Phe200				Glu202
	Asp178	1	Met201				
	Glu202	2	Ile203				
			Ile208				
HPrK/P-Compound 6	Ile158	1	Val141		Ser156	Lys160	Glu162
	Gly159	1	Ile158		Ser161	Arg204	Asp177
	Ser161	1	Ile203				Asp178
	Glu162	1					Glu202
	Asp178	1					
	Glu202	2					

**Table 3:** List of free binding energy (MM-GBSA) of the top six compounds with *M. pneumoniae* HPrK/P.

Complex Name	ΔVDWAALS (kJ/mol)	ΔE_EL_ (kJ/mol)	ΔE_GB_ (kJ/mol)	ΔE_SURF_ (kJ/mol)	ΔG_GAS_ (kJ/mol)	ΔG_SOLV_ (kJ/mol)	ΔTotal (kJ/mol)
HPrK/P-Compound 1	−22.56 ± 0.67	−15.39 ± 3.53	20.46 ± 0.70	−3.53 ± 0.01	−37.95 ± 3.59	16.92 ± 0.70	−21.03 ± 3.66
HPrK/P-Compound 2	−34.21 ± 1.22	−51.31 ± 6.29	63.73 ± 0.65	−5.02 ± 0.08	−85.52 ± 6.40	58.71 ± 0.66	−26.82 ± 6.44
HPrK/P-Compound 3	−45.95 ± 0.15	−92.41 ± 2.40	106.65 ± 1.05	−4.95 ± 0.02	−138.36 ± 2.41	101.70 ± 1.05	−36.66 ± 2.63
HPrK/P-Compound 4	−11.82 ± 0.00	−41.08 ± 4.25	48.81 ± 1.04	−2.69 ± 0.04	−52.90 ± 4.25	46.13 ± 1.04	−6.78 ± 4.38
HPrK/P-Compound 5	−35.11 ± 0.99	−35.70 ± 1.73	45.04 ± 0.05	−5.22 ± 0.01	−70.81 ± 1.99	39.83 ± 0.05	−30.99 ± 1.99
HPrK/P-Compound 6	−21.24 ± 0.85	−95.06 ± 1.15	83.72 ± 0.96	−3.49 ± 0.07	−116.30 ± 1.44	80.23 ± 0.96	−36.07 ± 1.73

## Data Availability

Data are contained within the article.

## Supplementary Materials

The following supporting information can be downloaded at: https://www.mdpi.com/article/10.3390/life14060657/s1, Figure S1: Sequences and structural alignment of mycoplasma HPrK/P and human Serine /Threonine protein Kinase.; Figure S2: The MD simulation (RMSD analysis) of HPrK/P-compound 1, HPrK/P-compound 2, HPrK/P-compound 3, HPrK/P-compound 4, HPrK/P-compound 5, HPrK/P-compound 6 complexes for 100 ns.; Figure S3: Radius of gyration (Rg) of the six complexes during the MD simulation process.; Figure S4: Number of hydrogen bonds of the six complexes during the MD simulation process.; Figure S5: The 2nd MD simulation (RMSD analysis) of HPrK/P-compound 3, HPrK/P-compound 5, HPrK/P-compound 5 complexes for 100 ns.; Table S1: Sequence information for multiple sequence alignment.; Table S2: List of ADME and toxicity analyses for the top 10 compounds.; Table S3: 2614 FDA-approved drugs.: Table S4: 948 bioactive small molecules.
