# Supplementary material for: Structure-Based Virtual Screening, ADMET Properties Prediction and Molecular Dynamics Studies Reveal Potential Inhibitors of Mycoplasma pneumoniae HPrK/P
Source: Life (Basel). 2024 May 22;14(6):657. doi: 10.3390/life14060657 (PMC11204831; doi:10.3390/life14060657)
Supplement: Supplementary file 1 [file life-14-00657-s001.zip › life-3007908-supplementary.pdf]

Supplementary Figures and Tables

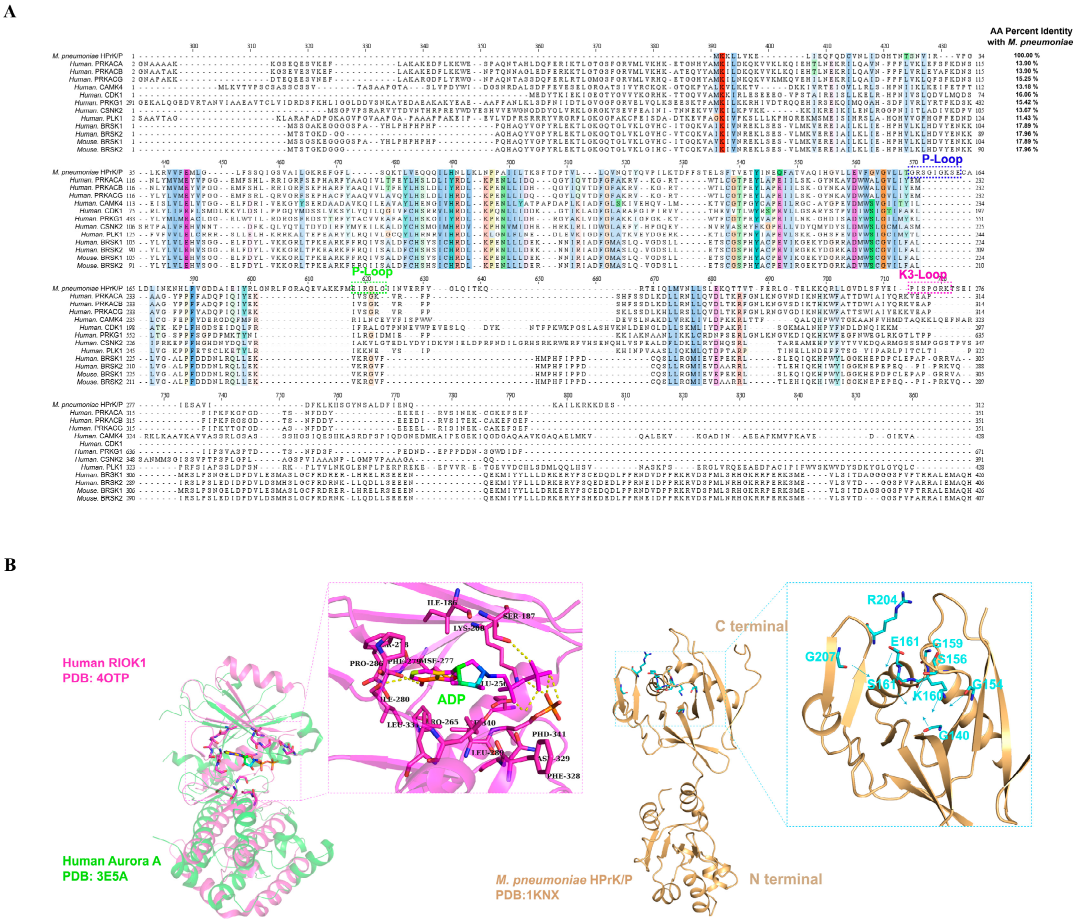

Figure S1. Sequences and structural alignment of mycoplasma HPrK/P and human serine/threonine protein kinase.

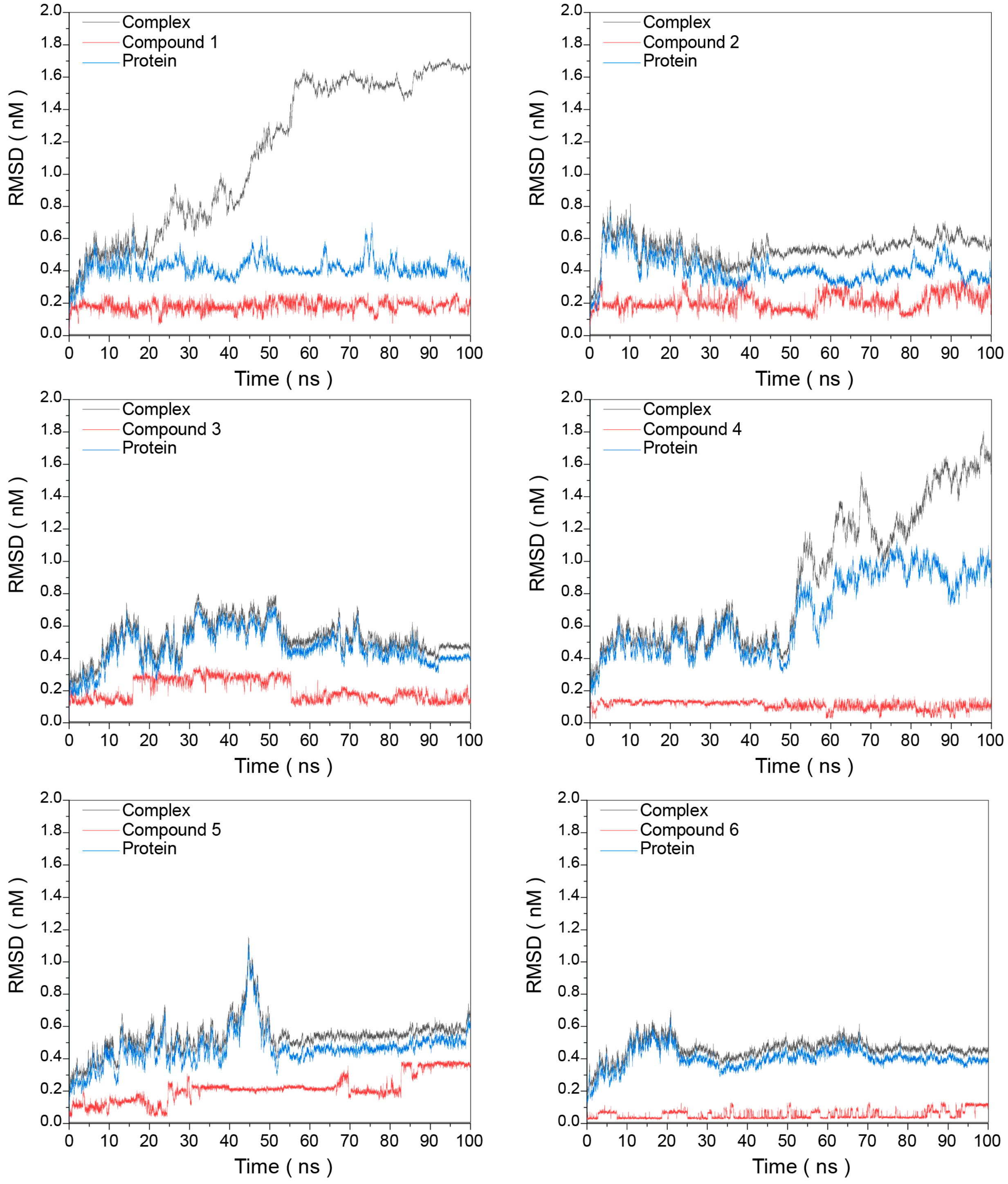

Figure S2. The MD simulation (RMSD analysis) of HPrK/P–compound 1, HPrK/P–compound 2, HPrK/P–compound 3, HPrK/P–compound 4, HPrK/P–compound 5,and HPrK/P–compound 6 complexes for 100ns.

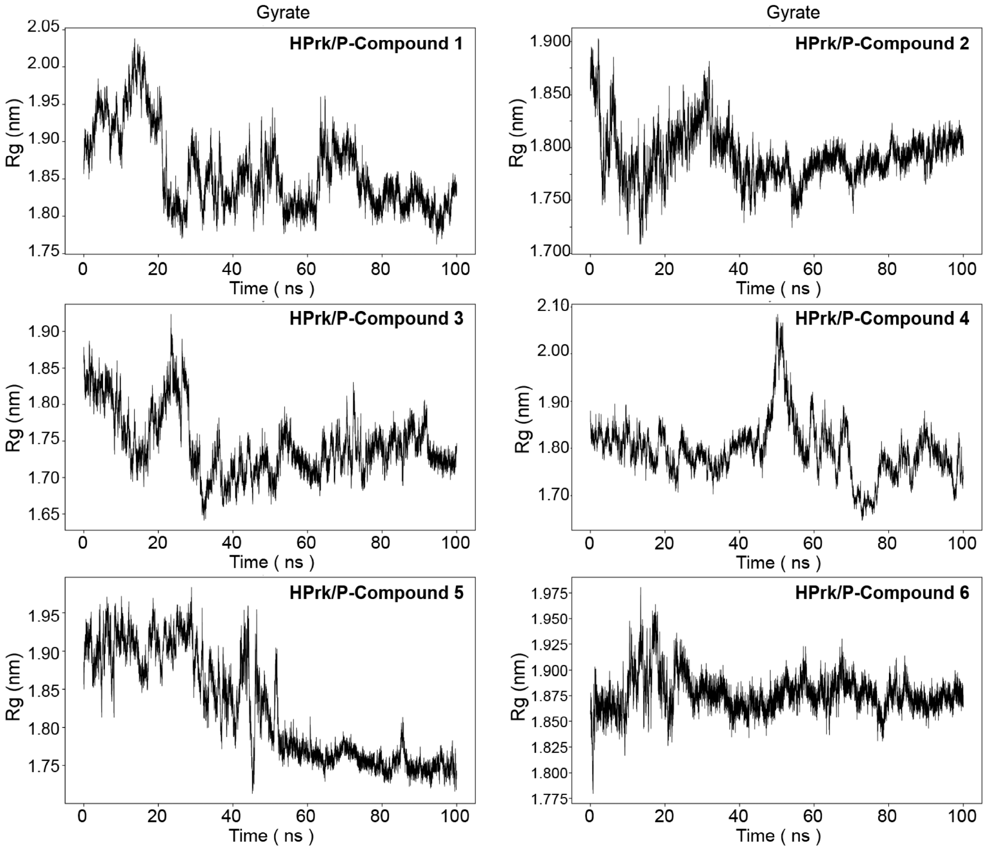

Figure S3. Radius of gyration (Rg) of the six complexes during the MD simulation process.

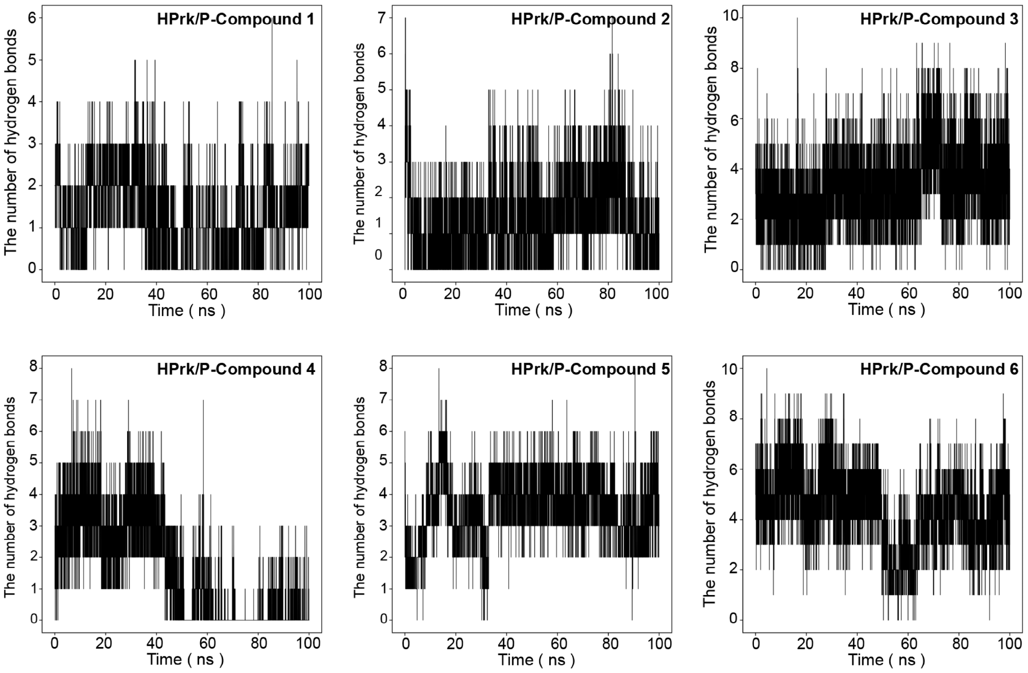

Figure S4. Number of hydrogen bonds of the six complexes during the MD simulation process.

Table S1. Sequence information for multiple sequence alignment.

Refer to the Attached Excel File Table S1.

Table S2. List of ADME and toxicity analyses for the top 10 compounds.

Molecule	Formula	Water Solubility	GI absorption	BBB permeant	Pgp substrate	TPSA	Log S	Log P	Log P	CYP1A2 inhibitor	CYP2C19 inhibitor	CYP2C9 inhibitor	CYP2D6 inhibitor	CYP3A4 inhibitor	log Kp (cm/s)	AMES Toxicity	Carcinogencity
**Compound 1**	C12H20N2O3	Soluble	High	No	Yes	85.61	-1.64	-0.25	0.15	No	No	No	No	No	-7.81	Non	Non
**Compound 2**	C15H22N6O5S	Soluble	Low	No	No	80.05	-3.94	2.38	2.25	No	No	No	No	No	-11.21	Non	Yes
**Compound 3**	C19H19N7O6	Moderately soluble	Low	No	No	213.28	-3.69	-2.21	-0.64	No	No	No	No	No	-9.76	Non	Non
**Compound 4**	C5H12N2O2	Soluble	High	No	No	89.34	0.40	-3.44	-1.62	No	No	No	No	No	-10.24	Non	Non
**Compound 5**	C18H21NO5	Moderately soluble	High	No	Yes	91.18	-3.07	1.42	1.81	No	No	No	Yes	No	-6.81	Non	Non
**Compound 6**	C6H10O6	Soluble	Low	No	No	107.22	0.17	-1.84	-1.48	No	No	No	No	No	-8.83	Non	Non
**Compound 7**	C17H21NO4	Soluble	High	No	Yes	92.95	-2.72	0.85	1.48	No	No	No	Yes	Yes	-6.76	Non	Non
**Compound 8**	C19H26I3N3O9	Soluble	Low	No	Yes	199.89	0.02	-2.31	-1.39	No	No	No	No	No	-13.47	Non	Non
**Compound 9**	C21H23N3O2	Poorly soluble	High	Yes	Yes	77.15	-3.29	2.28	2.03	Yes	No	No	Yes	No	-6.31	Yes	Non
**Compound 10**	C18H23NO4	Moderately soluble	High	No	Yes	92.95	-3.16	0.875	1.78	No	No	No	Yes	No	-7.57	Non	Non

Table S3. Depiction of 2614 FDA-approved drugs.

Refer to the Attached Excel File Table S3.

Table S4. Depiction of 948 bioactive small molecules.

Refer to the Attached Excel File Table S4.

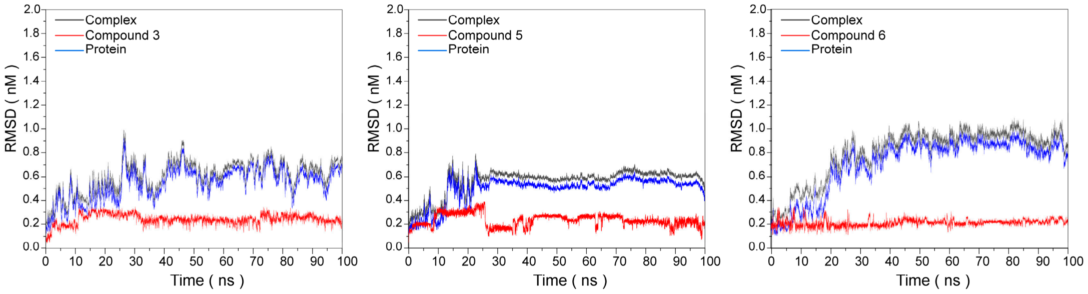

Figure S5. The second MD simulation (RMSD analysis) of HPrK/P – compound 3, HPrK/P – compound 5, and HPrK/P – compound 5 complexes for 100ns.
